# Ventilation through small‐bore airways in children by implementing active expiration

**DOI:** 10.1111/pan.14379

**Published:** 2021-12-22

**Authors:** Michiel de Wolf, Dietmar Enk, Narasimhan Jagannathan

**Affiliations:** ^1^ Department of Anesthesiology and Pain Medicine Maastricht University Medical Center Maastricht The Netherlands; ^2^ Medical Faculty University of Münster Münster Germany; ^3^ General Academic & Clinical Anesthesiology Ann & Robert H. Lurie Children's Hospital of Chicago Chicago Illinois USA

**Keywords:** airway, PICU, critical care, airway devices, devices, equipment, techniques

## Abstract

Management of narrowed airways can be challenging, especially in the smallest patients. This educational review focusses on active expiration through small‐bore airways with the Ventrain (Ventinova Medical, Eindhoven, The Netherlands). Manual ventilation with the Ventrain establishes inspiratory and expiratory flow control: By setting an appropriate flow, the volume of gas insufflated over time can be controlled and expiration through a small‐bore airway is expedited by jet‐flow generated suction, coined “expiratory ventilation assistance” (EVA). This overcomes the inherent risks of emergency jet ventilation especially in pediatric airway emergencies. Active expiration by EVA has been clinically introduced to turn a “straw in the airway” into a lifesaver allowing not only for quick and reliable reoxygenation but also adequate ventilation. As well as managing airway emergencies, ventilating through small‐bore airways by applying EVA implements new options for pediatric airway management in elective interventional procedures. Safe application of EVA demands a thorough understanding of the required equipment, the principle and function of the Ventrain, technical prerequisites, clinical safety measures, and, most importantly, appropriate training.

## 1 INTRODUCTION

Narrowed or shared airways (e.g., in ear‐nose‐throat surgery) can be encountered in pediatric anesthesia for several reasons including subglottic cysts, hemangiomas, or stenosis both in elective and in emergency settings.^1^ Also, the delicate laryngeal anatomy especially in newborns and babies and the small diameters of their airways increase the likeliness of injury following manipulations and subsequent significant swelling.

Different techniques are available for airway management in these patients, including intubation with a small‐bore airway like an intubating catheter (IC) or airway exchange catheter (AEC)^2^ or (in older children) transtracheal placement of a dedicated, kink‐resistant cannula.

Ventilation through these artificial airways requires specialized equipment as a decrease in their diameter increases resistance to flow exponentially. As a result, the necessary pressure gradient for both inspiration and expiration needs to be increased. The required high inspiratory pressure can be provided by gas (cylinder or wall) outlet pressure, but the pressures generated by the passive recoil of the lungs and chest wall are likely to be insufficient for exhalation in a timely manner. Consequently, these children will be either hypoventilated as expiratory time needs to be considerably prolonged, or air trapping will easily occur because of impeded expiration.

Use of the Ventrain can overcome these problems: It is a manually operated, flow‐controlled ejector ventilator and has been developed to assist the expiratory egress of gas by jet‐flow generated suction.^3^ Combined with a high‐pressure oxygen / air source, it can generate both the high pressure needed to overcome the resistance to inspiratory flow of a small‐bore airway and sufficient subatmospheric pressure to facilitate expiration through the same small‐bore cannula or catheter, while inspiratory tidal volume can easily be estimated from the set flow and inspiratory time. This process has been coined expiratory ventilation assistance (EVA).^4^ Introduced and initially evaluated in combination with a 2 mm inner diameter (ID) cricothyroidotomy cannula for adults,^3^, ^5^ the Ventrain has been applied in both emergent and elective clinical airway management. Generally, it can be used on any airway with a Luer lock, such as ICs, AECs, working channels of flexible or rigid bronchoscopes and (with some limitations) bronchial blockers.

Below, we will explain the mode of action of Ventrain, proper handling and operational requirements, efficiency of EVA, the correct use of EVA with mandatory safety measures, and the advantages over other means of ventilation through small‐bore airways. Finally, we will give some recommendations for using Ventrain / EVA in pediatric patients.

## 2 THE VENTRAIN

### 2.1 Mode of action

**FIGURE 1:**
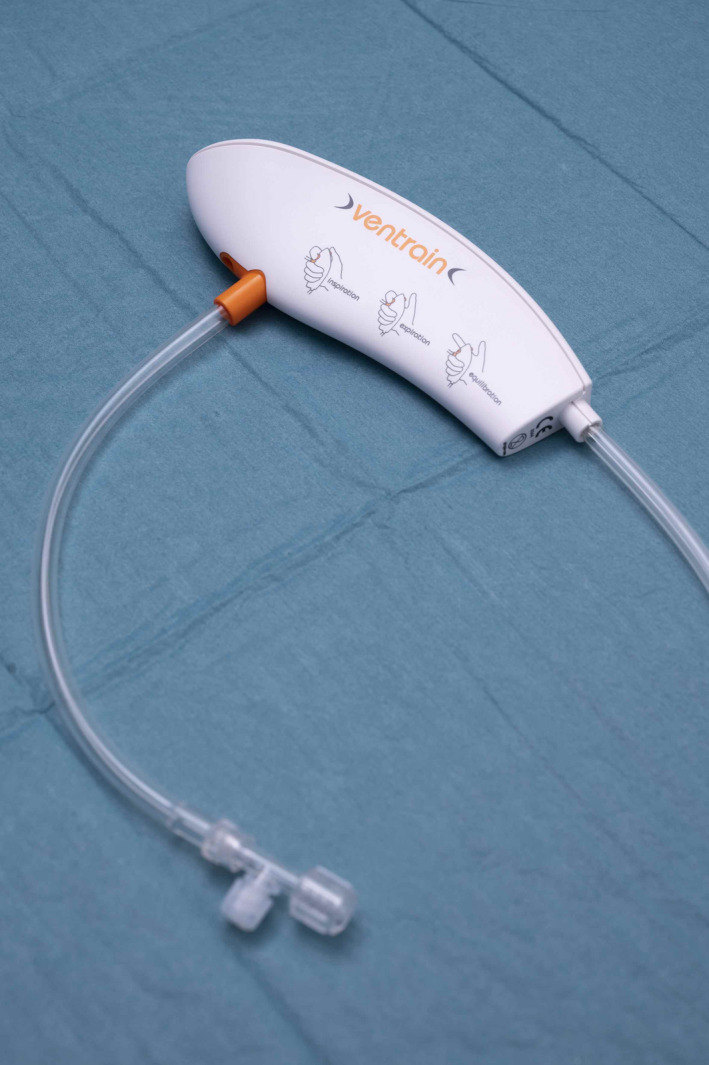
Ventrain

The Ventrain [Figure 1] is a single‐use hand‐held device that is directly connected to the outlet of a high‐pressure gas source (e.g., flow regulator of an oxygen cylinder, wall‐mounted flowmeter) by a 2 m long tubing. Located at the bottom of the Ventrain is a 20 cm long tubing with a distal T‐piece, which is attached to the patient's small‐bore airway via a Luer lock connector. Inside the shell is a purpose‐built ejector.

**FIGURE 2:**
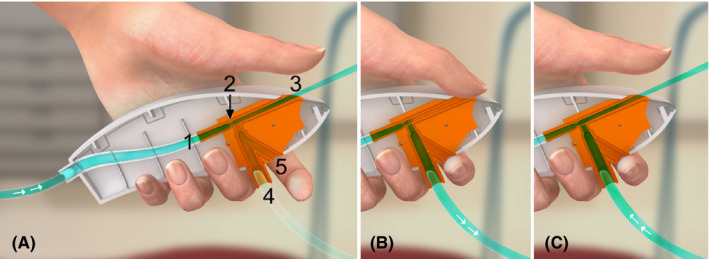
Handling of Ventrain. Gas flows from a high‐pressure source (1) through a 0.75 mm ID nozzle (2). (A) Equilibration: Releasing both the upper (3) and lower aperture (5) will result in slow equilibration of intrapulmonary and ambient air pressure. (B) Inspiration: By occluding both apertures simultaneously, gas will flow toward the patient (4). (C) Expiration: By releasing the upper aperture while keeping the lower occluded, the subatmospheric pressure created by Bernoulli's principle distally of the nozzle will result in active expiration (figure from^20^)

On its way from the high‐pressure oxygen source through the ejector, the gas passes a 0.75 mm ID nozzle [Figure 2]. As a result, the velocity and therefore the dynamic pressure (~ kinetic energy) of the flowing gas increase. This leads to a decrease in static pressure (~ potential energy) of the gas surrounding the jet released from the nozzle (Bernoulli's principle). During expiration, this resulting subatmospheric pressure is transmitted via the 20 cm long tubing to the patient's small‐bore airway and actively supports egress of respiratory gas.^3^

### 2.2 Handling and operation

After setting the desired flow, the Ventrain is manually operated by intermittently occluding and releasing one or two apertures [Figure 2]. The bottom aperture which is controlled by the operator's index finger acts as an on‐off‐switch. As long as this opening is released, the Ventrain is functionally disconnected (no airflow occurs to the patient, and, by excessively aspirating air from the environment, the subatmospheric pressure generated by the ejector is almost completely abolished). The top opening which is the exhaust of the ejector and controlled by the operator's thumb is the switch between inspiration and expiration.

If simultaneously closed [Figure 2B], the flow as set at the oxygen source is directed to the patient, whereas immediately after release of the top opening the ejector will aspirate respiratory gas from the patient via the small‐bore airway [Figure 2C]. Thus, inspiration demands closure of both openings at the same time, whereas active expiration is initiated by only releasing the top opening while keeping the bottom opening occluded. Releasing both openings switches the Ventrain off and allows (almost complete) pressure equilibration of the intrapulmonary with the environmental pressure [Figure 2A]. In other words, switching to equilibration mode immediately after insufflation of gas initiates a slow, passive expiration. Only occluding the top opening with the bottom opening released will result in some leakage noise without any relevant flow to the patient (=the device is then still functionally switched off).

### 2.3 Flow control

Contrary to hand‐held or automated (mechanical) jet ventilators, where inspiratory pressure is set but inspiratory volume can hardly be estimated, Ventrain is a flow‐controlled device, and inspiratory tidal volumes can therefore easily be estimated from the set flow and inspiratory time: If the flow is set to 6 L/min (=6000 ml in 60 s), then during inspiration 100 ml will be insufflated per second. Similarly, a flow of 2 L/min will result in an inspiratory tidal volume of 33 ml/s [Table 1].

**TABLE 1:** Flow set and volume administered per time

Flow (L/min)	Insufflated volume (ml) after 1 s inspiration	Insufflated volume (ml) after 2 s inspiration
2	33	66
3	50	100
6	100	200
9	150	300
12	200	400
15	250	500

With increased flow, suction pressure becomes more subatmospheric meaning also suction capacity (expiratory volume per time unit) will increase. When ventilating through a 7.5 mm long, 2 mm ID cricothyroidotomy cannula (for adults) in an ideal setting of no air leak, at flows of 9 L/min and lower, suction capacity will be slightly higher than inspiratory flow, whereas at higher flows the opposite applies.^3^

When ventilating adults through a cricothyroidotomy cannula, an inspiration / expiration (I:E) ratio approximating 1:1 is used. Most small‐bore airways used in pediatric patients (like an IC / AEC) will have a higher resistance to flow due to their decreased diameter and increased length compared to an adult‐sized cricothyroidotomy cannula. As most cylinder or wall oxygen / air sources deliver flow at a maximum pressure equal to or above 3.5 bar (50.8 psi), inspiratory flow will remain constant regardless of the higher resistance to flow. However, suction pressure generated within the Ventrain ejector is determined by the set flow only and will not increase at higher resistance to flow.

Therefore, when ventilating through long small‐bore catheters in high‐compliant lung models for benchmark testing, suction capacity will decrease and an I:E ratio of 1:1.5 up to 1:2 may be needed to prevent hyperinflation. However, the (very) low‐compliant chests of newborns, babies, and toddlers help for the expiratory egress of respiratory gas to some extent. In addition, in clinical practice at least some air leakage will occur around small‐bore catheters in most cases, so inspiratory tidal volume can be expected to be lower than estimated from the set flow. As leakage also supports expiratory egress of gas, an I:E ratio of 1:1 may still prove to be safe in the majority of pediatric patients during ventilation with Ventrain.

Even though inspiratory tidal volume can easily be calculated in a sealed airway, actual delivered volume into the patient's lungs will be less if leakage occurs. Increasing the flow or increasing inspiratory time may then be considered. Expiratory tidal volume can only be estimated as it is dependent on many factors (e.g., set flow, resulting subatmospheric pressure, resistance to flow, and leakage).

The minute volume can be calculated from the flow and the I:E ratio: In a sealed airway, a flow of 12 L/min at an I:E ratio of 1:1 will deliver a minute volume of 6 L/min. If an I:E ratio of 1:2 is needed, it will be 4 L/min (one third of 12 L/min).

### 2.4 Efficiency of reoxygenation and ventilation

Traditionally, insufflation or injection of gas through a small‐bore airway was only possible, if the ventilated patient's own airway was still (at least partially) open for passive exhalation to occur. In contrast, when applying EVA, reoxygenation within a few seconds and normoventilation can be achieved without the need for patency around the artificial airway. In fact, oxygenation and ventilation are most efficient with Ventrain in case of full airway occlusion.^6^ This is comparable to use of a regular endotracheal tube (ETT) where ventilation and oxygenation are improved by cuff inflation which minimizes leak, allows intrapulmonary pressure buildup and thus efficient alveolar gas exchange.

**FIGURE 3:**
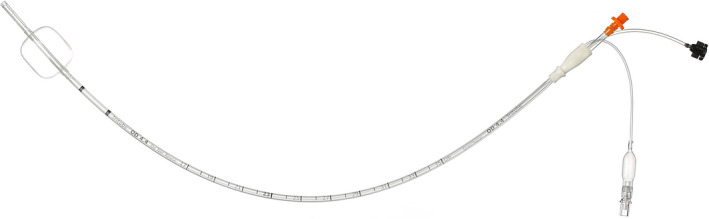
Tritube with inflated cuff (with courtesy of Ventinova Medical, Eindhoven, The Netherlands)

To this end, the Tritube (Ventinova Medical, Eindhoven, The Netherlands) was developed. It is a 45 (previously 40) cm long, 2.4 mm ID, 4.4 mm outer diameter (OD), cuffed ETT with three lumens—a ventilation lumen with a Luer lock connector, a channel allowing continuous tracheal pressure monitoring, and a lumen for elective cuff inflation to seal the airway [Figure 3]. As a comparison, the ODs of a 3.0 mm ID cuffed ETT (Covidien, Dublin, Ireland) and an 8 Fr and 14 Fr AEC (Cook Medical, Bloomington, IN, USA) are 4.2, 2.7, and 4.7 mm, respectively.

The Tritube has been designed to be used in combination either with the Ventrain [7; https://www.youtube.com/watch?v=‐qPPXiP0rVY&t=6s; accessed on 21st November 2021] or Evone (Ventinova Medical, Eindhoven, The Netherlands), a mechanical respirator applying the same principle as Ventrain but implementing pressure‐limited inspiration and full (=automatically adjusted) expiratory control. The current versions of the Tritube and Evone are approved for patients of 40 kg or more which limits their use in pediatric patients.

## 3 TECHNICAL PREREQUISITES

### 3.1 Pressure of the gas source

The auxiliary oxygen outlet of an anesthesia machine or a built‐in flowmeter should not be used for ventilation with Ventrain as most cannot generate the required pressure and may therefore deliver lower flows than set and displayed.^8^, ^9^

In addition, any high‐volume tubing (e.g., the anesthesia circuit) or humidifier reservoirs connected in‐line must be avoided, because they will act as a buffer and become pressurized (which is potentially harmful).

### 3.2 Requirements for flowmeters and flow regulators

The flow may only be set within the range of a flowmeter. A setting outside the scale can result in uncontrolled, excessively high flow.

Because Ventrain is a high‐pressure device, the gas source must be able to handle back pressure, which means to deliver a stable flow against (high) resistance to flow. Column flowmeters with a floating ball or cylinder inside (=rotameter) attached to a wall outlet can do this, but their flow display can become wrong if they are not pressure‐compensated (i.e., the column is calibrated for uncompressed gas). In these flowmeters, after connection to the Ventrain, the gas inside the column will be compressed because of the high pressure proximally of the ejector nozzle, leading to a drop of the floating ball or cylinder. Even though the flow is still as set before connection of Ventrain, an erroneously lower flow will be indicated. This should be ignored and not entrap the clinician to increase the flow as then a dangerously higher flow may be delivered to the patient. To change the flow, the Ventrain must be disconnected first. After reconnection, the floating ball or cylinder can be expected to drop again.

In pressure‐compensated flowmeters, the column is calibrated for compressed gas (the calibration pressure is usually indicated on the flowmeter's column) and the flow reading can be expected to be accurate when the Ventrain is connected. These two types of flowmeters can easily be distinguished by setting them at a flow and then partially occluding the outlet (and thereby creating some back pressure). If the indicated flow stays the same, the flowmeter is pressure‐compensated, whereas it is not if the flow reading drops.

Instead, to avoid confusion, an oxygen cylinder with a flow regulator dial is the better choice for the Ventrain. Flow regulators are typically less precise compared to flowmeters but can always handle the back pressure caused by Ventrain. Furthermore, an oxygen cylinder with a flow regulator dial and the Ventrain form a portable emergency ventilation system for applying EVA through small‐bore airways almost everywhere.

If the clinician wishes to be able to regulate the inspiratory oxygen concentration, gas from oxygen and air flowmeters or flow regulators may be blended. For safety reasons, only flowmeters of the same type (either pressure‐compensated or nonpressure‐compensated) should be combined.

One must bear in mind that oxygen from a cylinder or wall outlet is dry. Therefore, only short‐term use is advisable. For intermittent humidification of the mucous membranes during longer lasting ventilation with Ventrain, one may consider to slowly inject small increments of 0.1–0.2 ml of saline solution with a 1 ml syringe into the insufflated gas via the side port of the distal T‐piece (=the capnometry port, see below).

## 4 SAFETY CONSIDERATIONS AND MONITORING WHEN USING THE VENTRAIN

Training is essential to safely use the Ventrain and to efficiently apply EVA. In our experience, repeated training sessions are necessary as most clinicians will not use Ventrain regularly. With the help of a disposable glove, it is easy to become familiar with the equipment and to gain some experience [https://www.youtube.com/watch?v=LtQzeZJ6x4I; accessed on 21st November 2021]. Preferably, novices in this technique should be supervised by more experienced users, especially in pediatric patients.

If relatively more gas is insufflated over time, the intrapulmonary pressure will rise. If relatively more gas is aspirated, the intrapulmonary pressure will drop. Interestingly, subatmospheric intrapulmonary pressure does apparently not affect the lung tissue (e.g., intrapulmonary edema and/or bleeding) as has been shown in artificial coughing experiments in guinea pigs.^10^ However, subatmospheric pressure may cause edema of the mucous membrane in the trachea, so excessive expiration should be avoided to decrease the risk of subglottic swelling.

In a sealed airway, intrapulmonary pressure buildup may quickly lead to circulatory deterioration, particularly in neonates and infants. Plausibly, in a functionally open airway the risks resulting from a volume imbalance diminish.

As ventilation with the Ventrain is performed manually, inspiratory and expiratory time will vary. Furthermore, resistance of the artificial airway and the flow set influence efficiency of EVA, leading to differences in expiratory volume over time. These imbalances in inspiratory and expiratory volumes and, consequently, intrapulmonary pressure can be expected to become more pronounced if the operator is distracted.

Therefore, meticulous observation and clinical judgment of thorax excursion during inspiration and return during expiration are imperative when ventilating with the Ventrain. For safety reasons, 5 ventilation cycles should always be followed by an equilibration pause of at least 5 s.

Continuous tracheal pressure measurement during ventilation with the Ventrain requires a separate lumen as in the Tritube^7^ but its outer diameter of 4.4 mm plus the deflated cuff precludes use in children with narrowed airways. Intermittent measurement of intrapulmonary pressure^11^ can be done instead (as described below) during an equilibration phase after stabilization of the patient, so that the Ventrain operator is not distracted during ventilation.

### 4.1 Intermittent intrapulmonary pressure measurement

**FIGURE 4:**
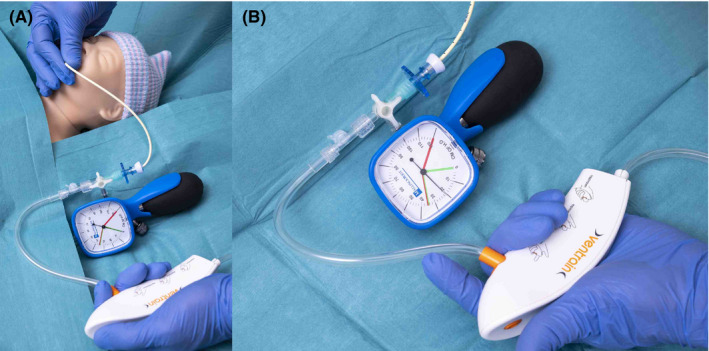
(A+B) Intermittent pressure measurement. (A) During ventilation, the connection to the manometer must be closed to prevent damage to the manometer. (B) For measuring end‐inspiratory (alveolar) pressure, both openings of the Ventrain are released at the end of insufflation. Now, the three‐way stopcock is turned so that only the manometer and the small‐bore airway are connected and the Ventrain is shut off. The same can be done at the end of (active) expiration to measure end‐expiratory (alveolar) pressure

Intermittent measurement of intrapulmonary pressure can be performed with the help of a three‐way stopcock and a manometer (e.g., a cuff pressure manometer which is unfortunately not certified for this purpose; [https://www.youtube.com/watch?v=XVLcoHavCJo; accessed on 21st November 2021]): First, a three‐way stopcock is attached in‐line between the distal T‐piece of the connecting tubing of Ventrain and the small‐bore airway. Then, the manometer (or its pressure measurement line) is connected to the side port of the three‐way stopcock [Figure 4A]. Reliable intrapulmonary pressure measurements can only be obtained in a static (=no flow) situation, because only then the pressure equalizes (=principle of communicating tubes). So, at the end of inspiration or expiration, after switching to equilibration mode (by releasing both apertures of Ventrain), the three‐way stopcock is turned so that it exclusively connects the manometer to the small‐bore airway (with the Ventrain shut off). Now, an immediate reading of intraalveolar pressure can be obtained [Figure 4B].

This way inappropriately high or low intrapulmonary pressure can be quickly corrected by adjusting the I:E ratio and/or flow.

After each measurement, the three‐way stopcock is turned again to exclusively connect the Ventrain to the small‐bore airway (with the manometer shut off).

In a dynamic situation (i.e., during ventilation with the Ventrain) with the manometer continuously switched on, measurements are not reliable because of the significant pressure drop across the small‐bore airway. Furthermore, the manometer may be damaged by the high‐pressure swing proximally of the small‐bore airway.

Most importantly, intermittent intrapulmonary pressure measurement can also provide information on air leakage and therefore patency of the patient's upper airway: If during an end‐inspiratory measurement the pressure is seen to decline, this is indicative of an air leak with the rate of decline as a measure of the patency of the patient's upper airway (assuming no pathologies leading to intrapulmonary loss of air are present). If, however, the measured intrapulmonary pressure remains constant, the upper airway is tightly sealed. The result of an intermittent pressure check can thus help the clinician in decision‐making, for instance on the possibility of changing the airway to an ETT with a larger diameter.

### 4.2 Intermittent capnometry

The distal T‐piece of the connecting tubing of the Ventrain has a side port (with a cap) to which the tubing of a side‐stream capnometer can be directly attached (due to regulatory issues this functionality is not available in the United States). End‐tidal capnometry (ETCO_2_) demands a slow, passive expiration (=switching to equilibration mode after an inspiration), because otherwise expiratory suctioning of the Ventrain interferes with aspiration of respiratory gas by the side‐stream capnometry pump.

Before reading the ETCO_2_ value, one must wait for the capnograph to reach a plateau (because of the dead space of the side‐stream capnometry system this will take several seconds) [https://www.youtube.com/watch?v=FW8mPyWOFBg; accessed on 21st November 2021]. After the measurement, ventilation is resumed. If the measurement indicates (increasing) hypoventilation, minute volume and thus the flow must be increased. If (increasing) hyperventilation is noted, the opposite applies. A change in frequency will not change the minute volume, as this is only determined by flow and I:E ratio.

In case of leakage, the reading will be less reliable and may thus only be used as a relative measure. Importantly, capnometry can help in confirming the small‐bore airway is placed intratracheally.

## 5 COMPARISON OF EVA TO OTHER OXYGENATION / VENTILATION TECHNIQUES

Use of anesthesia machines / circuits with large‐bore ventilation tubing in combination with small‐bore airways equal to and below a 2.0 mm ID may give the impression of adequately ventilating patients, even though they are severely hypoventilated. This is because during inspiration high inspiratory pressure mainly compresses the gas column in the anesthesia circuit, instead of insufflating air into the patient. Therefore, specialized equipment should be used when ventilating through small‐bore airways.

Jet ventilation (i.e., injection of gas at high pressure through a small‐bore catheter) is inherently dangerous if the upper airway is not sufficiently open, especially if manually controlled hand‐held devices are used. Mechanical (high frequency) jet ventilators controlling intrapulmonary pause pressure in between the injection cycles may reduce the risk of overinflation, but they simply stop if high pause pressure is detected. In adults, emergency jet ventilation has been shown to be very hazardous with high failure rate.^12^ Plausibly, its use is even more questionable in pediatrics.

Lung injury / barotrauma by high intrapulmonary pressure is not the only risk of emergency jet ventilation. Inadequate egress of gas during expiration will also lead to auto‐PEEP, which can be deleterious to the patient's circulation. If gas injection is continued despite inadequate expiration, intrapulmonary pressure will quickly rise to dangerous levels. Even if the injection pressure is reduced to 0.5 bar (=7.25 psi), it is still about four times higher than the mean blood pressure of a healthy adult (90 mmHg = 120 mbar = 1.74 psi). Of course, due to the pressure drop across the small‐bore airway, the set injection pressure will typically not become effective to the patient's lungs. However, if the upper airway is sealed, the injection pressure may cause an intrathoracic pressure buildup within a few cycles which exceeds the blood pressure by far and leads to a functional stop of circulation.

Intermittent intrapulmonary pressure measurement (e.g., with the help of a three‐way stopcock and a manometer, see above) during manual jet ventilation only provides limited safety as a quick reduction of intrapulmonary pressure to restore circulation is impossible in case of an insufficiently open upper airway and lack of active expiration.

Another pitfall of emergency jet ventilation is the principle of pressure control: The injection pressure must be set and then, following release of the trigger, an unforeseeable flow into the patient's lungs results. Here, it becomes obvious why it is more appealing to rely on flow control rather than pressure control, because this allows for estimating the maximum gas volume insufflated over time (Table 1).

Inspiratory flow control can be easiest established by the principle of so‐called flow splitting which allows to switch between (full) flow as set at the gas (oxygen) source and almost no flow to the patient by manual closure and release of openings, respectively.

The use of a three‐way stopcock or another self‐assembled flow splitting device is inherently dangerous (not even considering medicolegal issues): While application of the full flow set by intermittently occluding one opening (e.g., the side port of a three‐way stopcock having a functional diameter of 2–3 mm) is appealingly easy, releasing such a small opening does not efficiently switch off the flow splitter.^13^ Therefore, in this setup, even if the side port is open to ambient air, a patient is at high risk of hyperinflation, so this technique should no longer be recommended.^14^, ^15^

Passive exhalation demands a significantly larger opening or multiple openings. The oxygen flow modulator (OFM; Cook Inc.) has five 4‐mm openings (which can only be completely occluded by an intentional maneuver) and its successor, the Rapid‐O2 (Meditech Systems Ltd.), has one large opening. Both establish a low resistance to expiratory flow. These devices only allow a slow, passive expiration driven by elastic recoil of the lung‐chest system (comparable to the equilibration phase in Ventrain). In case of a highly or completely obstructed upper airway, the small‐bore airway mainly defines the resistance to outflow of respiratory gas. Consequently, adequate expiration without relevant auto‐PEEP demands time (even in the noncompliant lung‐chest system of babies and small children) which necessarily results in hypercapnia because of inevitable hypoventilation over time.

In a bench study using adult, child, and infant airway models with varying degrees of proximal airway obstruction, it was found that of all tested devices only the Ventrain could consistently insufflate acceptable tidal volumes and avoid high airway pressures. The other devices variably led to hyperinflation, especially in infant lungs and in obstructed upper airways.^16^ Considering limited hypoventilation tolerance due to reduced functional residual capacity and undesired effects of prolonged and/or increased (auto‐)PEEP on circulation, EVA / active expiration becomes even more important in newborns, babies, and small children as it can provide a higher alveolar gas turnover and preservation of circulatory function.

## 6 USE OF VENTRAIN IN PEDIATRIC PATIENTS

### 6.1 Setting the correct flow and I:E ratio

A flow of 1 L/min per year of age (or the nearest flow selectable at the flowmeter / flow regulator) with a minimum of 2 L/min and a maximum of 15 L/min is set. Ventilation is started with an inspiratory time of 1 s (0.5 s, if <3 kg) and an I:E ratio of 1:1.

Alternatively, the flow and inspiratory time can be set according to (normal) weight of the patient to create a tidal volume of 6–10 ml/kg (refer to [Table 1]). Calculation of tidal volume may not be easy in a stressful emergency setting. Instead of memorizing Table 1 for future reference, one may keep in mind that a flow of 6 L/min will deliver 100 ml/s. Other flows can then easily be calculated from this, for instance 2 L/min will provide 33 ml/s.

If leakage occurs (which will happen in most instances), it may be necessary to increase the flow and/or inspiratory time as actual delivered tidal volume will be smaller than calculated tidal volume.

Meticulous observation of chest movement may help to adjust the I:E ratio appropriately: Visible return of the chest (and/or the upper abdomen) during the equilibration phase following expiration of the fifth ventilation cycle indicates intrapulmonary pressure buildup during ventilation, so active expiration should be prolonged during ventilation with Ventrain.

Commence intermittent measurement of intrapulmonary pressure (see above) to avoid significant imbalance in inspiratory and expiratory volumes.

Table 2 provides a quick reference guide on use of the Ventrain in pediatric patients.

**TABLE 2:** Preferred setup and clinical application of the Ventrain

Connect the long tubing of Ventrain to the flow regulator of an oxygen cylinder. Connect the T‐piece of the short tubing to an intratracheally placed cannula or catheter with Luer lock. Set a flow of 1 L/min per year of age (minimum 2 L/min, maximum 15 L/min). Alternatively, calculate desired tidal volume based on the flow set (refer to Table 1). Start ventilating by simultaneously occluding both apertures with an inspiratory time of 1 s (0.5 s, if <3 kg). Switch then to expiration by only releasing the upper aperture. Continue ventilation at an inspiration / expiration (I:E) ratio of 1:1. Meticulously observe thorax excursion and its return. Every 5 cycles at the end of expiration release both apertures (=equilibration mode) for at least 5 s. Visible return of the chest and / or the upper abdomen during the equilibration phase indicates intrapulmonary pressure buildup during ventilation, so active expiration should be prolonged when resuming ventilation with Ventrain. Set up intermittent measurement of intrapulmonary pressure as soon as possible. Consider intermittent capnometry. Evaluate flow and I:E ratio (e.g., higher flow or longer inspiration in case of air leak to obtain an adequate tidal volume, longer expiration time may be needed in case of complete upper airway obstruction). If sufficient oxygenation and ventilation are established, start planning the next step in managing the airway.

### 6.2 Experience so far in pediatric patients

Ventrain's instructions for use state that it can be used for “all patients, however, for patients with body mass <40 kg (e.g., children, infants) [it] should only be used for lifesaving maneuvers.” Therefore, experience in pediatrics is limited and based on expert opinion. So far, to our knowledge, in children the Ventrain has mostly been used for ventilation through 8 Fr intubating catheters (ICs) or airway exchange catheters (AECs).

The first description of its use in infants is on two babies of 2.1 and 4.3 kg planned for surgery who could not be conventionally intubated with a 2.5 mm ID ETT despite good laryngeal view because of laryngeal swelling. In both cases, bag mask ventilation became increasingly difficult, so an 8 Fr (2.66 mm OD, 1.66 mm ID) IC (35 cm length) and AEC (45 cm length), respectively, were placed. Oxygenation and ventilation were quickly reestablished with Ventrain, buying time for intubation with an ETT as soon as laryngeal swelling subsided.^17^

Another case report tells how a 3.7 kg baby developed acute respiratory failure a day after laryngeal surgery. Rigid bronchoscopy was performed, revealing significant glotto‐subglottic edema. During bronchoscopy, ventilation was accomplished by connecting the Ventrain to the working channel of the bronchoscope. After intubation with a 2.0 mm ID ETT, ventilation with the PICU ventilator was inadequate due to high pressures but possible with Ventrain.^18^

Use of AECs has been described in the management of neonates and infants with laryngeal stenosis where an ETT could not be placed due to its size.^2^ Although oxygenation / ventilation (either manually assisted or mechanically assisted) through these catheters was possible in 10 patients of 2.1–3.4 kg, an emergency tracheostomy had to be performed in one patient of 2.6 kg due to inadequate oxygenation. Oxygen insufflation and jet ventilation through AECs have led to serious morbidity and mortality in adults, because continuous insufflation or intermittent injection of oxygen without appropriate pressure limitation and/or adequate exhalation necessarily result in gas trapping.^19^ Quite likely, gas trapping will also occur during elective ventilation through an AEC if the stenosis around the catheter impedes expiration. If instead efficient bidirectional flow through these catheters is accomplished with Ventrain, this complication can be prevented.

**FIGURE 5:**
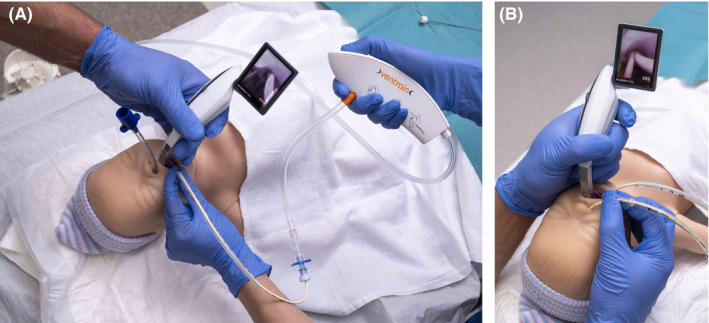
Use of Ventrain during tube exchange (A). After placing a new endotracheal tube (ETT) nasally, an 8 Fr airway exchange catheter (AEC) already connected to the Ventrain is placed through the oral ETT (shortened and tube connector removed to facilitate tube exchange) and oxygenation / ventilation through the AEC with Ventrain is started. The oral ETT is then withdrawn to ease intubation with the nasal ETT. (B) If the orotracheal AEC hinders atraumatic placement of the nasal ETT, first a nasally advanced IC or AEC (with the new ETT already slided on) can be placed intratracheally. Then, oxygenation / ventilation with Ventrain is switched from the orotracheal to the nasotracheal IC / AEC, and the orotracheal AEC is withdrawn. Now, the nasal ETT can be railroaded over the nasotracheal IC / AEC

Ventrain can also prevent an emergency situation or desaturation: We have repeatedly used the Ventrain in combination with ICs / AECs during ETT exchange to continuously oxygenate pediatric patients at risk for desaturation even in short periods of apnea or with a previous difficult intubation. Typically, these patients were orally intubated, but a nasal tube was requested to facilitate weaning: For quick and safe exchange, the length of the oral ETT should preferably be less than half the length of the AEC. Therefore, it is prudent to shorten the oral ETT before starting the procedure. First, the patient is preoxygenated, and the nasal ETT is placed in the nasopharynx. An AEC (with the Luer lock connector and Ventrain already attached and the desired oxygen flow set) is placed through the oral ETT to be exchanged. The depth of the AEC should be the same (or only slightly deeper) as the oral ETT to prevent endobronchial positioning and/or mucosal injury. Ventilation with the Ventrain is started by a second operator [Figure 5A]. Air leakage may be expected as the proximal end of the oral ETT is open. Under (video)laryngoscopic view the oral ETT can now be withdrawn while the AEC is kept in position and manually fixated in the corner of the patient's mouth by the Ventrain operator. Preferably, the oral ETT is left over the AEC allowing quick repositioning just in case. The nasal ETT is now further advanced by the anesthetist, and the patient intubated. Because desaturation will not occur as quickly as in apnea (however, as air leakage is likely, oxygenation and ventilation may not be optimal) time is won for intubation with the nasal ETT, and, if necessary, advanced techniques like fiberoptic inspection / guidance can be facilitated. After confirmation of tracheal intubation and correct positioning of the nasal ETT, the AEC can be withdrawn. As the larynx can become quite crowded if an ETT is placed alongside an AEC, it may sometimes be necessary to first intubate with a nasally advanced IC or second AEC [Figure 5B]. After the nasal ETT has been slided on the catheter, ventilation with the Ventrain can be switched and continued via the nasotracheal catheter. Now, under (video)laryngoscopic view, the orotracheal AEC can be withdrawn before the nasal ETT is railroaded over the nasotracheal catheter.

## 7 REFLECTIVE QUESTIONS

1. Which equipment do we have at my institution for pediatric patients with narrowed airways, and where can I find it?
2. In elective surgery / interventions in critical pediatric airways (e.g., laryngeal stenosis), how will I secure the airway and provide safe ventilation?
3. Do I know the technical prerequisites for (emergency) ventilation with the Ventrain, how to choose an appropriate oxygen flow and to adapt inspiratory and expiratory time in different pediatric age groups?
4. What are the safety measures while applying EVA?

## CONFLICT OF INTEREST

M. de Wolf has no conflict of interest to declare. D. Enk is the inventor of the Oxygen Flow Modulator and EVA / FCV technology (Ventrain, Tritube, Evone). He receives royalties for the Oxygen Flow Modulator from Cook Medical and royalties for EVA / FCV technology (Ventrain, Tritube, Evone) from Ventinova Medical. He is a paid consultant to Ventinova Medical. N. Jagannathan serves on the editorial boards of Anesthesia and Analgesia, Pediatric Anesthesia, Journal of Clinical Anesthesia, and Journal of Anesthesia. He has received products free of charge from Ambu and Teleflex. He has received travel support for meetings involving future developments for upcoming airway devices from Teleflex, Vyaire Medical, and Mercury Medical. He has received stock options from Spiro.

## Data Availability

Data sharing is not applicable to this article as no new data were created or analyzed in this study.
